# Detection of Antibodies Against Tick-Borne Encephalitis Virus and Other Flaviviruses in a Zoological Collection in Slovenia

**DOI:** 10.3389/fvets.2021.688904

**Published:** 2021-06-24

**Authors:** Pavel Kvapil, Joško Račnik, Marjan Kastelic, Pavlína Pittermannová, Tatjana Avšič-Zupanc, Eva Bártová, Kamil Sedlák

**Affiliations:** ^1^Ljubljana Zoo, Ljubljana, Slovenia; ^2^Department of Biology and Wildlife Diseases, Faculty of Veterinary Hygiene and Ecology, University of Veterinary Sciences Brno, Brno, Czechia; ^3^Institute of Poultry, Birds, Small Mammals and Reptiles, Faculty of Veterinary Medicine, University of Ljubljana, Ljubljana, Slovenia; ^4^Faculty of Medicine, Institute of Microbiology and Immunology, University of Ljubljana, Ljubljana, Slovenia; ^5^Department of Virology and Serology, State Veterinary Institute Prague, Prague, Czechia

**Keywords:** West Nile virus, Usutu virus, tick-borne encephalitis virus, blood sampling, zoo animals

## Abstract

Monitoring infectious diseases is one of the most important pillars of preventative veterinary medicine in zoological collections. The zoo environment offers a great variety of different animal species living in proximity and in contact with small wild animals and vectors (e.g., ticks and mosquitos). In this context, tick-borne encephalitis virus (TBEV), Usutu virus (USUV), and West Nile virus (WNV) causing vector-borne diseases are emerging pathogens that raise concern. The aim of the study was to detect antibodies to selected flaviviruses in various animal species in the Ljubljana Zoo, Slovenia. In total, 874 sera from 96 animal species were tested for antibodies to TBEV by enzyme-linked immunosorbent assays (ELISA); positive samples were confirmed by a virus neutralization test (VNT) using TBEV, WNV, and USUV antigens. Antibodies to TBEV were detected by ELISA in 3.9% (34/874) of zoo animals, with 4% (30/753) in mammals and 5% (4/86) in birds; the sera of reptiles (*n* = 34) and amphibians (*n* = 1) were negative. Antibodies to TBEV were confirmed by VNT in 11 mammals; one bird was positive for both WNV and USUV. The mixture of exotic animal species and their contact with wild animals and vectors such as ticks and mosquitos suggest that screening of infectious diseases in zoo animals might provide good insight into the epizootological situation of the area. This is the first survey of TBEV, WNV, and USUV in a zoological collection in Slovenia.

## Introduction

Zoos are areas where different exotic animal species live in proximity, usually in contact with wild animals as well as humans (zookeepers and visitors). Monitoring infectious diseases is one of the most important parts of veterinary care in zoological collections in order to discover possible emerging pathogens and to provide safety for animals and visitors. The mixture of various hosts, reservoirs, and pathogens can be utilized as a sentinel for screening for emerging infectious diseases (1–3). Tick-borne encephalitis (TBE) is one of the most important flaviviral human diseases in Europe and Asia. The causative agent TBE virus (TBEV) has a positive singlestranded RNA genome and is a member of the tick-borne flavivirus group (genus *Flavivirus*, family *Flaviviridae*) (4, 5). TBEV is transmitted by the bite of an infected tick or, rarely, through raw milk products from infected mammals (6). The virus circulates between vector ticks and some of their hosts, mostly deer and small mammals such as rodents and insectivores, whereas only small mammals are presumed to be competent virus reservoir hosts (5). In Europe, TBE is one of the most common flavivirus infections of the central nervous system and is endemic to several countries. Slovenia is among European countries with the highest reported TBE incidence rates (8.1–18.6 cases/100,000 population in the past decade) (7). Accumulated evidence has underlined the emerging potential of TBEV, how virus variants are selected, adapted to the tick vector and rodent host, with possible major implications for clinical disease. TBEV has a huge geographic distribution, including at least 34 countries (8). TBEV is the most important causative agent of arboviral infection in Europe, causing neurologic symptoms. The incidence of the disease has greatly increased over the past decades, and in the meantime, some changes in spatial distribution of TBE cases have been observed. Therefore, it is important to recognize the distribution of endemic areas, to use preventive measures. Molecular results indicate that the virus can be detected in the organs of the rodents for longer periods, indicating prolonged infections of the rodent hosts by the virus. Rodents can therefore be used as a useful indicator of the circulation of TBEV in an area (9). Some arboviruses from the family *Flaviviridae—*for example, TBEV—have been present in Slovenia for many years (10). Other viruses, such as West Nile virus (WNV) and Usutu virus (USUV), were introduced only recently (11). The aim of this study was to screen for TBEV antibodies in zoo animals and based on its cross-reactivity detect some other possible flaviviruses (WNV, USUV).

## Materials and Methods

### Blood Sampling and Serological Examination

The study took place at the Ljubljana Zoo, which is the only large collection of zoo animal species in Slovenia. Between 2006 and 2018, it was home to between 480 and 560 animals belonging to 130–160 species. During those years, all samples obtained from routine clinical examinations were stored and frozen. All clinical procedures have been performed according to standard operation protocols (SOP) considering human and animal safety. Altogether, 874 serum samples of 96 animal species were tested for antibodies to TBEV with enzyme-linked immunosorbent assays (ELISA, EIA TBEV Ig, TestLine Clinical Diagnostic, Brno, Czech Republic). The examination was performed according to the manufacturer's instructions. All ELISA-positive samples were confirmed by a virus neutralization test (VNT) in micromodification, with vital staining by method according to the method described previously (12). Briefly, a 100 μl working dilution of virus was mixed and incubated with an equal portion of the test serum and added to the same portion of the cell culture suspension. The plates were incubated in 5% CO_2_ at 37°C. VNT was performed with TBEV (Strain Hypr) and concurrently with WNV (WNV Strain Line 2) and USUV (Austrian strain). Viruses were grown on the brains of sucking lab mice at 1–3 days of age. Suspension of porcine kidney cell line (PS) was used as a cell substrate for TBEV VNT. Suspension monkey kidney cell line (CV-1) was used as a cell substrate for WNV VNT and USUV VNT. Working dilution for both cell lines was 600,000 cells/ml. Serum toxicity control was included in the tests, as well as specific positive and negative control sera. The result of VNT is a virus neutralization (VN) titer, which is the reciprocal of the highest sample dilution that is still capable of neutralizing the cytopathic effect at more than 50% (TCID50). Samples were marked as positive if the VN titer > 4 (12).

## Results

Antibodies to TBEV were detected by ELISA in 3.9% (34/874) of zoo animals, with 4% (30/753) in mammals, 5% (4/86) in birds, and 0% in reptiles (*n* = 34) and amphibians (*n* = 1). Antibodies to TBEV were found by VNT in 10 serum samples (one Alpine ibex, four domestic sheep, four mouflons, and one red fox). Antibodies to WNV and USUV were found by VNT in a barn owl (*Tyto alba*), with a titer of 128 positive for both viruses. Other samples were negative. The results are summarized in Table 1.

**Table 1:** Results of serological examination of zoo animals for antibodies to tick-borne encephalitis virus (TBEV) tested by enzyme-linked immunosorbent assays (ELISA) with confirmation of positive samples by virus neutralization test (VNT).

**English name**	**Latin name**	**ELISA (positive/tested)**	**VNT (positive/tested)**	**TBEV titers**
**MAMMALS**		**30/753**		
Alpaca	*Vicugna pacos*	1/34	Neg.	
African hedgehog	*Atelerix albiventris*	0/2		
Alpine ibex	*Capra ibex*	5/121	1/5	32
Asian elephant	*Elephas maximus*	0/6		
Bactrian camel	*Camelus bactrianus*	0/32		
Bank vole	*Myodes glareolus*	0/7		
Black-and-white ruffed lemur	*Varecia variegata*	0/2		
Black-tufted marmoset	*Callithrix penicillata*	0/1		
Brown bear	*Ursus arctos*	0/5		
Brown rat	*Rattus norvegicus*	0/14		
Capybara	*Hydrochoerus hydrochaeris*	0/3		
Cattle	*Bos taurus*	0/7		
Chamois	*Rupicapra rupicapra*	0/3		
Chapman's zebra	*Equus quagga chapmani*	0/7		
Chimpanzee	*Pan troglodytes*	0/9		
Chinchilla	*Chinchilla lanigera*	0/1		
Dog	*Canis lupus familiaris*	0/1		
Domestic goat	*Capra aegagrus hircus*	2/35	Neg.	
Domestic pig	*Sus scrofa domesticus*	0/4		
Domestic rabbit	*Oryctolagus cunicullus*	0/7		
Domestic sheep	*Ovis aries*	5/54	4/5	8,16,16,64
Donkey	*Equus asinus*	0/2		
Fat dormouse	*Glis glis*	0/13		
Eurasian badger	*Meles meles*	0/2		
European water vole	*Arvicola amphibius*	0/20		
Fallow deer	*Dama dama*	2/59	Neg.	
Reticulated giraffe	*Giraffa camelopardalis reticulata*	0/3		
Golden hamster	*Mesocricetus auratus*	0/6		
Gray wolf	*Canis lupus*	0/4		
Guanaco	*Lama guanicoe*	0/8		
Guinea pig	*Cavia porcellus*	0/2		
European hedgehog	*Erinaceus europaeus*	0/1		
Horse	*Equus caballus ferus*	0/9		
Indian crested porcupine	*Hystrix indica*	0/1		
Kafue lechwe	*Kobus leche kafuensis*	0/3		
Lesser hedgehog tenrec	*Echinops telfairi*	0/3		
Lion	*Panthera leo*	0/1		
Long-tailed field mouse	*Apodemus sylvaticus*	0/32		
Eurasian lynx	*Lynx lynx*	0/4		
Meerkat	*Suricata suricatta*	0/18		
Moose	*Alces alces*	1/3	Neg.	
Mouflon	*Ovis aries musimon*	7/58	4/7	TS
Western European house mouse	*Mus musculus domesticus*	0/22		
Northwestern wolf	*Canis lupus occidentalis*	0/2		
Patagonian mara	*Dolichotis patagonum*	0/22		
Persian leopard	*Panthera pardus saxicolor*	0/2		
Red deer	*Cervus elaphus*	4/7	Neg.	
Red fox	*Vulpes vulpes*	2/2	1/2	16
Red panda	*Ailurus fulgens*	0/2		
Reindeer	*Rangifer tarandus*	0/3		
California sea lion	*Zalophus californiensis*	0/2		
Siberian tiger	*Panthera tigris altaica*	0/1		
Squirrel monkey	*Saimiri bolliviensis*	0/6		
Wild boar	*Sus scrofa*	1/3	Neg.	
Wild rabbit	*Oryctolagus cuniculus*	0/6		
Yellow-cheeked gibbon	*Nomascus gabriellae*	0/11		
Yellow-necked field mouse	*Apodermus flavicollis*	0/53		
Zebu	*Bos taurus indicus*	0/2		
**BIRDS**		**4/86**		
African gray parrot	*Psittacus erithacus*	0/2		
Barn owl	*Tyto alba*	1/3	Neg.	
Black stork	*Ciconia nigra*	0/4		
Black-crowned night heron	*Nycticorax nycticorax*	0/11		
Blue-and-yellow macaw	*Ara ararauna*	0/1		
Domestic chicken	*Gallus gallus domesticus*	0/1		
Common buzzard	*Buteo buteo*	0/2		
Common cuckoo	*Cuculus canorus*	0/2		
Hooded crow	*Corvus cornix*	0/6		
Domestic goose	*Anser anser domesticus*	0/12		
Emu	*Dromaius novaehollandiae*	0/3		
Eurasian eagle-owl	*Bubo bubo*	0/2		
Eurasian griffon vulture	*Gyps fulvus*	1/6	s.s.	
Eurasian tree sparrow	*Passer montanus*	0/2		
Green-winged macaw	*Ara chloropterus*	0/1		
Gray heron	*Ardea cinerea*	0/3		
Helmeted guineafowl	*Numida meleagris*	0/4		
Salmon-crested cockatoo	*Cacatua moluccensis*	0/1		
Common ostrich	*Struthio camelus*	0/5		
Great white pelican	*Pelecanus oncrolatus*	1/7	s.s.	
Rose-ringed parakeet	*Psittacula krameri*	0/1		
Snowy owl	*Bubo scandiacus*	1/1	s.s.	
Mute swan	*Cygnus olor*	0/1		
Turquoise-fronted amazon	*Amazona aestiva*	0/1		
Ural owl	*Strix uralensis*	0/3		
**REPTILES**		**0/34**		
African spurred tortoise	*Centrochelys sulcata*	0/1		
Bearded dragon	*Pogona vitticeps*	0/1		
Corn snake	*Pantherophis guttatus*	0/1		
European pond turtle	*Emys orbicularis*	0/2		
Green iguana	*Iguana iguana*	0/2		
Hermann's tortoise	*Testudo hermanni*	0/1		
Panther chameleon	*Furcifer pardalis*	0/1		
Indian python	*Python molurus*	0/2		
Red-eared slider	*Trachemys scripta elegans*	0/3		
Sulcata tortoise	*Centrochelys sulcata*	0/2		
Yellow-bellied slider	*Trachemys scripta scripta*	0/18		
**AMPHIBIANS**		**0/1**		
Balkan frog	*Pelophylax kurtmuelleri*	0/1		

*s.s., small sample*.

## Discussion

Flaviviruses have become increasingly important pathogens in Europe over the past few decades. TBEV, which causes central nervous system infections in humans, is causing health problems in Europe and Asia. This virus is mainly transmitted via tick bites, however, raw milk products might also be a source of infection (13, 14). In 2018, a total of 3,092 cases of TBE were reported in EU countries (15). In recent years, TBE has emerged in previously unaffected regions (e.g., the Netherlands), and the number of cases has doubled in Slovakia, Lithuania, and Croatia (15). Because of the lack of effective treatment for TBE, anti-TBE immunization and the avoidance of tick bites are of key importance in preventing this infection (16).

The incidence of TBEV in Slovenia was monitored by RT-PCR between 2005 and 2006 in ticks and humans (9). The overall prevalence of TBEV in 4,777 ticks collected in those 2 years was 0.47%. In a similar study from neighboring Croatia, a 1.1% rate of TBEV was found in ticks and 1.6% in spleen samples from wild fox (*Vulpes vulpes*) and red deer (*Cervus elpahus*) (17). Furthermore, the infection rate detected in ticks by RT-PCR significantly correlated with the TBEV incidence in humans in selected areas. The sequencing method proved the genetic correlation between TBEV in collected ticks and Slovenian patients infected with TBEV. A study performed on rodents during the same period showed an average of 5.9% TBEV antibody prevalence detected by immunofluorescence assay in various Slovenian regions (9). The results varied by rodent species and trapping region. The overall prevalence was comparable with the results in our study (3.9%). Surprisingly, 0% prevalence was detected in 82 samples from three rodent species (*Myodes glareolus, A. flavicollis*, and *A. sylvaticus*) in our study. This may be explained by the high correlation of infection rate depending on selected areas, mentioned in the studies above. This could further suggest that the high variability of sentinel species enhances the possibility of identifying a targeted pathogen. In a similar study on rodents performed in Switzerland in 2006 and 2007, sera from 333 rodents were examined with a very similar overall prevalence of 3.9%. In some areas, the prevalence reached 9.9%, whereas in others the prevalence was 0%, as it was in our study. Serum samples from 1,014 wild boar and 758 roe deer were screened for flavivirus antibodies, using a competitive ELISA (cELISA) technique between 2009 and 2014 in France (18). A significantly higher incidence of flaviviruses was found in boar (5.6%) than in roe deer (2.1%). The distribution was also higher than expected in comparison to human cases. This finding confirmed the potential usefulness of wildlife in monitoring infectious diseases. In the Czech Republic, serum samples from 133 animals of 69 different animal species from five zoological collections located in various regions were evaluated to detect antibodies to TBEV (19). Two ungulates from the same zoo were seropositive in an area endemic for TBEV. This suggests that monitoring infectious diseases endemic in screened areas plays a crucial role in choosing the proper hot spot for placing sentinel animals to increase the chance of pathogen interception. This point has also been highlighted in another study (1). Results in our study have also shown, that the 9 of 10 TBEV-positive serum samples in VNT assay were found in Alpine ibex, domestic sheep and mouflons, which are closely related. Screening of infectious diseases with zoonotic potential is an important activity for preventive health care for the human and animal population. Tick-borne encephalitis virus is endemic in Slovenia, whereas West Nile virus and Usutu virus are pathogens that were only very recently introduced into the Slovenian region (11). In our study, samples from only one animal, a barn owl (*Tyto alba*), were positive for antibodies for WNV as well as for USUV. It seems that there was simultaneous coinfection with both viruses because there was a high VNT titer (128) for both viruses. Moreover, the positive sample was collected in autumn 2018, which was the year when both viruses (WNV and USUV) were introduced into Slovenia, with the first confirmed cases in humans and animals. Although USUV in particular is known to cause clinical disease and death in sensitive birds, such as owls, the positive barn owl showed no clinical signs and was still alive 26 months after the positive sample collection. Also, when looking at the dynamics of seroprevalence, the samples in our study were collected from 2006 to 2018, with 33 out of 34 TBE positive samples being detected from 2014 to 2018. During the last 2 years of our study, 24 samples tested positive, which could suggest the spread of the endemic infection into the hitherto TBEV-naive area. A follow-up comparative study on rodents and zoo animals in the near future could highlight the usefulness and importance of various sentinel animals for monitoring zoonotic diseases. The discrepancy between some of our TBEV ELISA and TBEV VNT results could be the result of the small sample volume, which is a common problem when obtaining samples during routine clinical procedures. The presence of WNV in Europe has been known for decades. Virus transmission is related to ornithophilic mosquitoes. Wild birds are an important natural amplifying host for the virus, whereas humans, horses, and some other mammals are considered dead-end hosts. Serological evidence in songbirds in Slovenia has shown a 3.7% prevalence of WNV antibodies in samples collected, detected by indirect immunofluorescence methods (20–22). In a PCR study on samples from birds of prey and owls from Slovenia collected between 1995 and 2013, no positive birds were found (22). In a recent study from Slovenia, a pool of *Culex* sp. mosquitos was found to be positive by RT-PCR in 2018, but negative in 2017 and 2019. A sentinel study on dog sera samples confirmed WNV antibodies by an indirect immunofluorescence study in 1.8 and 4.3% of samples collected in 2017 and 2018, respectively (23).

Considering the zoonotic potential of the aforementioned viruses, routine surveillance and screening of zoological collections on a regular basis could be an option for sentinel animal species' enhancement. The suitability of zoological collections as epidemiological stations has been discussed in the past (1–3). Sentinel animal species will be susceptible to disease, with rapid seroconversion, and will remain monitored by a supervising authority (1). Zoo animals cover a vast range of different species, with varying susceptibility to disease. In the case of WNV and USUV, wild birds are the principal hosts and mosquitos are the principal vectors. However, WNV has also been isolated from other mammals; for example, *Apodemus flavicollis, Clethrionomys glaerolis*, sentinel mice and hamsters, *Lepus europaeus*, camels, horses, dogs, and humans in enzootic foci (24). This suggests that choosing the proper hot spot for placing sentinel animals is crucial for increasing the chance of pathogen interception (1).

Screening of USUV in birds from four different zoological collections in Switzerland, Austria, and Hungary showed different results based on the geographic location and related mortality. In Switzerland and Vienna, three different zoos showed a prevalence of 5–9% for the presence of USUV antibodies, whereas all animals tested at a Hungarian zoo were serologically negative (25). Despite the fact that TBE, WNV, and USUV pathogens are closely related, their dynamics and therefore sentinel targeted screening are vastly different. Ticks are vectors for TBEV. Their regional prevalence as well as steady epidemiological situations in certain loci are typical. USUV and WNV, transmitted by mosquitos, have caused occasional outbreaks with unprecedented dynamics, probably connected with climate change and other factors, and slow introduction into countries such as Slovenia, where both diseases were not endemic. These specific conditions make unique settings, such as zoos with a variety of animals with known histories, good veterinary care, and established monitoring of infectious diseases in all taxa, suitable epidemiological stations for screening emerging pathogens. In a recent study, 120 various mammal species from 10 different Spanish zoological collections were assessed for flavivirus exposure between 2002 and 2019. Using similar methods, the presence of flavivirus was detected in 3.3% of the samples examined (3).

Our study was based on a simple serological survey of TBEV presence in zoo animals. However, considering our results and the literature data presented, further possible conclusions and future studies with an impact on human and animal health should be considered, in particular the role of zoos as possible epidemiological stations.

## Data Availability Statement

The original contributions presented in the study are included in the article/supplementary material, further inquiries can be directed to the corresponding author/s.

## Ethics Statement

The animal study was reviewed and approved by all samples were collected as a secondary interest by zoo personal during clinical procedures, surgeries, and annual health checkups, or from rodents included in pest control. No ethical approval was issued. All procedures involving animals were approved by the National Ethical Committee and the Administration of the Republic of Slovenia for Food Safety, Veterinary, and Plant Protection (Permit number 34401-7-2016-5, 31 January 2017). Animal care and treatment were conducted in accordance with the institutional guidelines and international laws and policies (Directive 2010/63/EU on the protection of animals used for scientific purposes).

## Author Contributions

PK was responsible for conceptualization, investigation, data curation, writing-original draft preparation, and writing-review and editing. JR was responsible for conceptualization, writing-original draft preparation, review and editing, investigation, and supervision. MK was involved in software, project administration, and visualization. PP was responsible for methodology, investigation, and data curation. TA-Z was involved in conceptualization, validation, software, and writing-review and editing. EB was responsible for conceptualization, methodology, software, resources, writing-original draft preparation, review and editing, supervision, and funding acquisition. KS was involved in methodology, validation, formal analysis, and writing-review and editing. All authors read and approved the final version of the manuscript.

## Conflict of Interest

The authors declare that the research was conducted in the absence of any commercial or financial relationships that could be construed as a potential conflict of interest.

## Abbreviations

s. s: small sample
TS: toxic serum.
